# Atypical Presentation of Takotsubo Cardiomyopathy in an Elderly Woman

**DOI:** 10.14797/mdcvj.1100

**Published:** 2022-06-24

**Authors:** Priya Arunachalam, Nina Manian, Lamees Ibrahim El Nihum, Saleem Toro, John M. Buergler

**Affiliations:** 1 Texas A&M College of Medicine, Bryan, Texas, US; 2 Houston Methodist DeBakey Heart & Vascular Center, Houston, Texas, US

**Keywords:** Takotsubo cardiomyopathy, apical ballooning syndrome, pericardial effusion, cardiac tamponade, hemodynamic instability

## Abstract

We describe an 83-year-old woman who presented to the emergency department with extreme thirst. Diagnostic testing revealed Takotsubo cardiomyopathy. Following symptomatic improvement and discharge, she returned to the emergency department with exudative pericardial effusion and elevated intrapericardial pressures. This case illustrates the importance of close follow-up of Takotsubo patients in whom complications such as pericardial effusion may lead to cardiac tamponade and hemodynamic instability if not managed properly.

## Background

Takotsubo cardiomyopathy, also known as apical ballooning syndrome and stress cardiomyopathy, is a rare syndrome associated with transient akinesis of the heart’s apical walls with refractory hyperkinesis of the basal wall.^1^ It is a diagnosis of exclusion. Common presenting symptoms of Takotsubo cardiomyopathy mimic myocardial infarction and include chest pain and dyspnea, reported in 67.8% and 17.8% of patients, respectively.^2^ Palpitations, nausea, vomiting, syncope, headache, epigastric pain, weakness, and hypotension also have been reported as initial presenting symptoms of Takotsubo cardiomyopathy.^3^ Though the pathophysiology is largely unknown, Takotsubo cardiomyopathy is commonly associated with physical and emotional stress. Electrocardiogram may show ST-segment elevation, T-wave abnormalities, or Q waves. Serum studies generally demonstrate elevated troponins. Echocardiogram is useful in making the diagnosis and will show evidence of apical akinesia with basal hyperkinesia, and coronary angiography is also useful to exclude significant coronary obstruction.

## Presentation

An 83-year-old woman presented to the emergency department (ED) with extreme thirst. She described an unquenchable thirst along with an ominous feeling that began the previous night. She denied shortness of breath, palpitations, chest pain, lower extremity edema, and recent stressors or illnesses. She had a history of chronic lower extremity deep vein thrombosis, diastolic congestive heart failure, hypothyroidism, and asthma. Echocardiogram 2 years prior showed a normal ejection fraction (EF).

## Investigations

**Figure 1:**
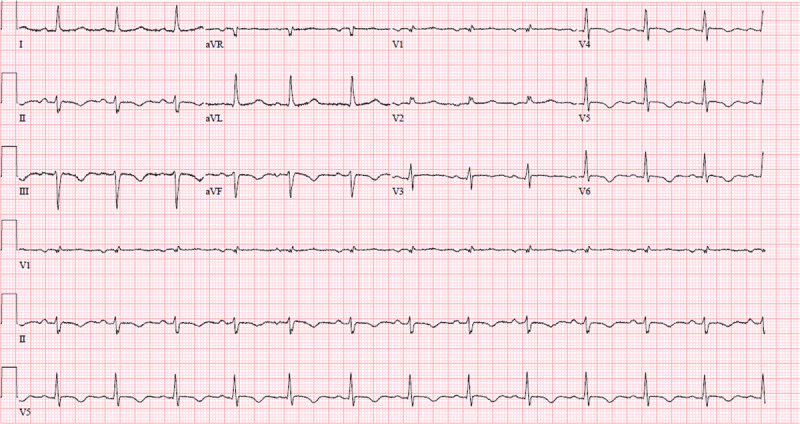
Presenting electrocardiogram showed T-wave inversions in the inferior leads.

On presentation, serum studies were significant for a troponin level of 4.241 ng/mL and a brain natriuretic peptide level of 201 pg/mL. Electrocardiogram showed T-wave inversions in the inferior leads (Figure 1). Echocardiogram demonstrated an EF of 30%.

During evaluation in the ED, the patient developed chest pain. After nitroglycerin administration, she became hemodynamically unstable and required pressors to maintain blood pressure. Repeat electrocardiogram was significant for diffuse T-wave inversions. Due to concern for non-ST-elevation myocardial infarction, she was immediately rushed to cardiac catheterization.

**Figure 2:**
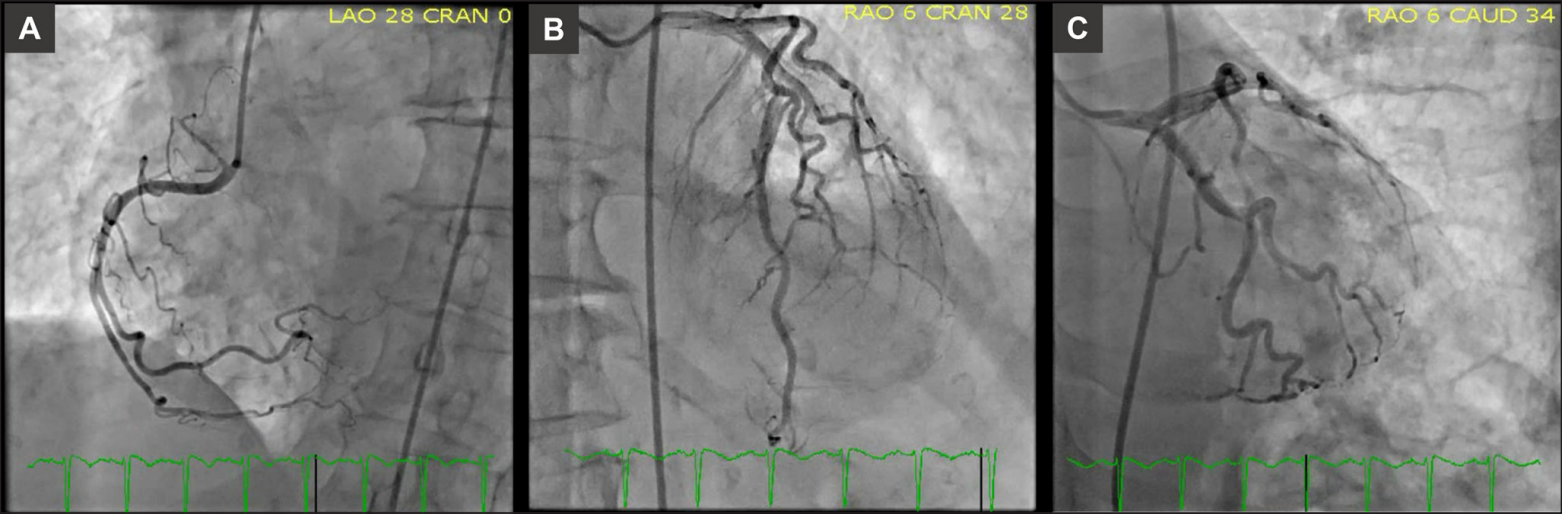
Coronary artery catheterization revealed a **(A)** dominant right coronary artery, **(B)** left anterior descending artery with no flow-limiting lesions, and **(C)** left circumflex artery and obtuse marginal branch with no flow-limiting disease.

**Figure 3:**
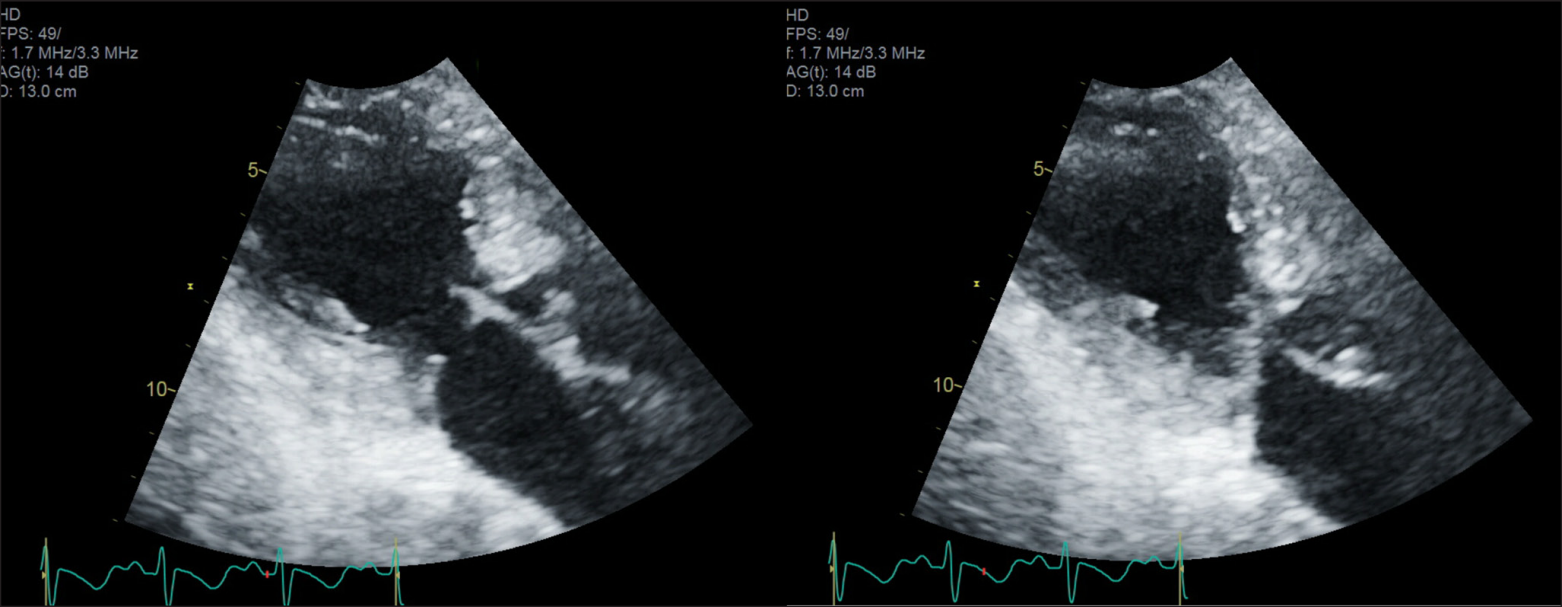
Echocardiogram demonstrated left ventricular outflow tract dynamic obstruction of 85 mm Hg.

Catheterization demonstrated no significant coronary artery disease (Figure 2) but did show significant left ventricular to aortic pullback gradient, suggesting Takotsubo cardiomyopathy-related left ventricular outflow tract obstruction (LVOT). Dynamic gradient was seen as well as an LVOT resting gradient of 20 mm Hg at baseline. Because of her severely elevated left-sided filling pressures in the setting of stress-induced cardiomyopathy, an EF of 30%, and hypotension, we elected to manage her with cautious diuresis and intra-aortic balloon pump (IABP) placement, with planned IABP removal the next morning. Echocardiogram confirmed the diagnosis of Takotsubo cardiomyopathy with an LVOT dynamic obstruction of 85 mm Hg and systolic anterior motion of the mitral valve (Figure 3; Video 1).

**Video 1:** Echocardiogram of parasternal long axis view showing systolic anterior motion of the mitral valve, also at https://youtu.be/EkWdKnCE_zI.

## Management

Her condition improved after initiation of beta blocker and diuretic therapy, and repeat echocardiogram 5 days after admission showed recovered EF with no resting LVOT obstruction. Prior to discharge, the patient developed chest pain and shortness of breath with hypoxia, and her troponin level was normal. Chest X-ray and computed tomography angiography (CTA) of the chest were significant for moderate pericardial effusion and bilateral pleural effusions. Her symptoms improved with diuresis, and she was discharged.

Six days after discharge, the patient presented to the ED after a syncopal episode when standing up from the toilet. Her blood pressure at the scene was 60/40. Her pressures gradually improved to a systolic pressure of 100, but the patient reported heavy chest pressure and was readmitted. CTA showed large pericardial effusion that was increased in size from her previous visit. Echocardiogram confirmed large pericardial effusion and demonstrated increased intrapericardial pressure and no inferior vena cava collapse. At this time, the patient was not in tamponade clinically. Diagnostic pericardiocentesis was significant for exudative pericardial effusion per Light’s criteria with negative cultures. She also had an elevated erythrocyte sedimentation rate.

The patient was initiated on pericarditis therapy, including high-dose aspirin and colchicine. Repeat echocardiogram showed stable loculated pericardial effusion with features consistent of constrictive pericarditis. A previously placed pericardial drain showed minimal drainage. She was evaluated for a pericardial window procedure, but the loculated nature suggested the effusion was not likely to be drained. Due to consistently up-trending inflammatory markers, daily steroids were added to the pericarditis treatment regimen. Echocardiogram prior to discharge showed elevated filling pressures with a small posterolateral pericardial effusion. Following hemodynamic stability and normalized inflammatory markers, she was discharged with plans for outpatient cardiology follow-up. At 3 months, the patient was doing well with resolution of her symptoms.

## Discussion

Many cases of classical Takotsubo cardiomyopathy have been reported in the literature. These cases largely report presenting symptoms of chest pain and/or dyspnea succeeding physical or emotional stress. Uncommon presenting symptoms include palpitations, nausea, vomiting, syncope, headache, epigastric pain, weakness, and hypotension as reported in reviews of existing literature.^1^ While several groups have proposed criteria for diagnosing Takotsubo cardiomyopathy, there are no universally accepted diagnostic criteria.^4^ Here, we present a case of Takotsubo cardiomyopathy with the unusual presentation of thirst as the initial symptom, followed by chest pain, which has not previously been reported in the literature. The pathophysiology of this complaint as it relates to Takotsubo cardiomyopathy may be a source of future study.

The prevalence of systolic anterior motion of the mitral valve and LVOT obstruction in Takotsubo cardiomyopathy is approximately 25%.^5^ Due to accelerated blood flow across the LVOT as a result of increased contractility, inotropes lead to increased systolic anterior motion of the mitral valve, thereby worsening LVOT obstruction. Therefore, the management of systolic anterior motion of the mitral valve and dynamic LVOT obstruction in patients with Takotsubo involves eliminating the use of inotropes and the prompt removal of the intra-aortic balloon pump to decrease the rate of flow across the LVOT. In addition, appropriate management involves increasing peripheral vascular resistance and afterload via direct vasopressors. Diuretics should be used cautiously due to the subsequent reduction of preload and afterload that also lead to worsening of LVOT obstruction.

Post-Takotsubo cardiomyopathy pericardial effusions and pericarditis are seen in up to 43% of patients.^3^ However, most patients are asymptomatic and do not present with a pericardial effusion large enough to elevate intrapericardial pressures, as seen in our patient. There are few case studies that document an exudative pericardial effusion leading to tamponade physiology.^6^ While the exact etiology of Takotsubo cardiomyopathy-associated pericarditis is unknown, one hypothesis is that the myocardial inflammation secondary to Takotsubo cardiomyopathy can spread to the pericardium and induce pericarditis, as seen in our patient. The first documented case of acute pericarditis following Takotsubo cardiomyopathy was seen in 2007, and the patient’s pericarditis was almost immediately ameliorated with a course of nonsteroidal anti-inflammatory medications.^7^

## Conclusion

Physicians should consider Takotsubo cardiomyopathy in the differential diagnosis of patients presenting with symptoms similar to those of myocardial infarction or in patients with elevated troponins and nonclassical symptoms such as nausea, vomiting, syncope, weakness, and thirst. This diagnosis should especially be considered in postmenopausal women regardless of absence of recent physical or emotional stress. Further, patients recovering from Takotsubo cardiomyopathy should be followed closely due to possible complications such as large pericardial effusions that, if not managed properly, could lead to cardiac tamponade and hemodynamic instability.

## References

[1] Sanchez-Jimenez EF. Initial clinical presentation of Takotsubo cardiomyopathy with-a focus on electrocardiographic changes: A literature review of cases. World J Cardiol. 2013 Jul 26;5(7):228-41. doi: 10.4330/wjc.v5.i7.228 23888192 PMC3722420

[2] Gianni M, Dentali F, Grandi AM, Sumner G, Hiralal R, Lonn E. Apical ballooning syndrome or takotsubo cardiomyopathy: a systematic review. Eur Heart J. 2006 Jul;27(13):1523-9. doi: 10.1093/eurheartj/ehl032 16720686

[3] Eitel I, von Knobelsdorff-Brenkenhoff F, Bernhardt P, et al. Clinical characteristics and cardiovascular magnetic resonance findings in stress (takotsubo) cardiomyopathy. JAMA. 2011 Jul 20;306(3):277-86. doi: 10.1001/jama.2011.992 21771988

[4] Wittstein I. Clinical presentation of takotsubo syndrome. In: Camm JA, Lüscher TF, Maurer G, Serruys PW, editors. ESC CardioMed (3 ed.) [Internet]. New York (NY): Oxford University Press; 2018 Dec [cited 2022 May 31]. Available from: https://oxfordmedicine.com/view/10.1093/med/9780198784906.001.0001/med-9780198784906-chapter-318

[5] El Mahmoud R, Mansencal N, Pilliére R, et al. Prevalence and characteristics of left ventricular outflow tract obstruction in Tako-Tsubo syndrome. Am Heart J. 2008 Sep;156(3):543-8. doi: 10.1016/j.ahj.2008.05.002 18760139

[6] Nagamori Y, Hamaoka T, Murai H, et al. Takotsubo cardiomyopathy complicated by cardiac tamponade due to non-hemorrhagic pericardial effusion: a case report. BMC Cardiovasc Disord. 2020 Feb 6;20(1):67. doi: 10.1186/s12872-020-01377-5 32028901 PMC7006067

[7] Maruyama T, Hanaoka T, Nakajima H. Acute pericarditis in the recovery phase of transient left ventricular apical ballooning syndrome (takotsubo cardiomyopathy). Intern Med. 2007;46(22):1857-60. doi: 10.2169/internalmedicine.46.0184 18025768

